# Skateboarding and the surplus value of city play

**DOI:** 10.3389/fspor.2024.1454274

**Published:** 2024-11-01

**Authors:** Brian Glenney, Isaac Bjorke, Andrea Buchetti

**Affiliations:** ^1^ Department of Global Humanities, Philosophy Program, Norwich University, Northfield, VT, United States; ^2^ Department of Economics, Columbia University, New York, NY, United States; ^3^ Department of History, Anthropology, Religions, Sapienza University of Rome, Rome, Italy

**Keywords:** play, urban, skateboarding, sport, leisure, deviance, labor, edgework

## Abstract

Cities, defined materially by concreted surfaces and geometrically shaped structures, have a novel ecology, a “grey space”. Grey spaces are criticized for their lack of salubrity in contrast to blue and green spaces enriched by natural biodiversity. How might cities become salubrious? We consider urban play as a source of surplus value both in the context of capitalist frames of labour vs. leisure and societal frames of obedience vs. deviance. We also discuss how some skate play is more ineffable, such as play that is for its own sake, deep play, edgework, and Promethean play. We explore these various facets of skate play in three spatial settings: (1) City-built skateparks, (2) DIY skateparks, and (3) Street spots. We then consider the more ineffable forms of skate play in the context of a Marxist framework of unalienated labour and argue that its unique reimaging of banal urban architecture: stairs, curbs, ledges, etc. creates a diversity of surplus value in the city. These more ineffable forms of play provide unique potential for human fulfilment and identity creation. Grey spaces can be enriched by social play diversity if cities open spaces for citizens to comfortably and naturally initiate diverse frames of play.

## 1 Introduction

**Figure 1:**
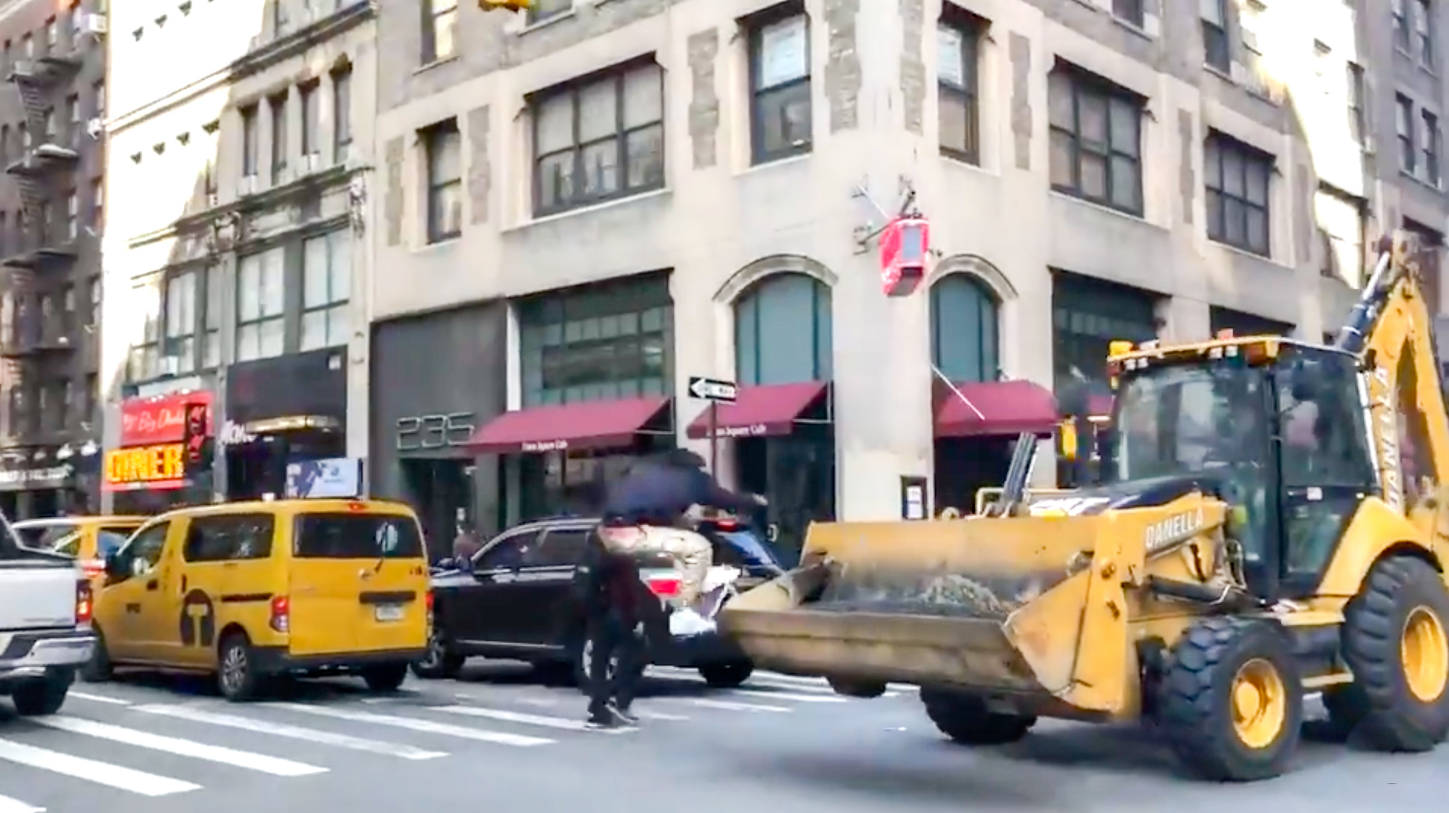
Professional skateboarder Tyshawn Jones plays dangerously in the streets of New York City with an impromptu trick on the front bucket of a backhoe that has just stopped.

Professional skateboarder, Tyshawn Jones, manoeuvres down a busy New York City Avenue, dodging approaching cars as he performs two “grinds” along the side of the street's thigh high ledges (1). Tyshawn then skates directly into an intersection, with cars slowing and stopping as they observe him approach. Suddenly, to Tyshawn's right comes a large yellow backhoe truck with its front bucket fast approaching. In a matter of seconds, we see the backhoe halt and Tyshawn jumps on the backhoe's front loader bucket—Tyshawn ollies up to perform a frontside boardslide on the loader bucket with his skateboard—nearly missing a surprised pedestrian as he lands (see Figure 1). Tyshawn then disappears into the stopped traffic as the videographer, Lui Elliott, laughs.

Tyshawn's manoeuvres exemplify a skateboarder's street play: revealing an exchange of risk and reward in its co-optation of urban space. By “manoeuvres”, we do not just mean a mere trick, but a “state of flow…’unthinking’ that unites—in a sudden movement—years of practice and hard work with the navigation of an immediately present physical environment” (2). The objects of Tyshawn's play, which includes ledges, stairs, handrails, and other urban architecture, are neither constructed for play, nor would they be thought of as objects of play by pedestrians. Rather, urban space, defined by its concreted surfaces and modern geometrically shaped structures, presents as a “grey” space, often defined by its dysbiotic ecology in contrast to salubrious green and blue spaces (3).

Grey space, commonly criticized for its lack of salubrious effects, from its lack of natural biodiversity (3), is the space of skateboarding (4). Attempts at greening urban spaces with parks, fountains, and street trees are complicated by a lack of the biodiversity otherwise found naturally in blue and green spaces (5). Grey space is thus idealized as out of balance and unnatural despite its increasing density—an unliveable clime. And yet, grey space is undergoing massive expansion, with 68% anticipated urban dwellers of a nearly 10 billion population by 2050 (6). How might urban space find some salubrity given its lack of biodiversity?

In this paper, we consider the social function and welfare benefits of play in the city as a stimulus for creating and reproducing “grey diversity”. This political economic analysis of play is but one of a plurality of strategies, complementing more qualitative ethnographic methods in sociology and anthropology. On this analysis, grey diversity is not achieved through the diversity of organisms in the soil, but rather is achieved through social and craft-like activity, where participants learn how to carve diverse uses of concreted materials prevalent in urban space (7). Some of this possibility has been recognized in work of polluted leisure in grey space (8, 9). Others have observed an economic market value to the city through tourism (10), as well as an increase in non-market value of social capital for individual users and sponsoring companies (11). We follow this latter observation, arguing that it is not merely the diversity of the material but the symbolically diverse way that a material space is used that provides this social value to the city. We argue that grey diversity is best exemplified when technologies for machines, i.e., urban architecture, becomes the technology of other tools, like skateboards. Skateboarding's candidacy for city play is due not just to its inhabiting grey space, but its nature: “Skateboarding is, at the most basic level, a form of playing” (2).

Grey play can be seen all over public urban spaces, such as purpose-built Playful Learning Landscapes (PLLs) like seesaws and swings. Montreal's “21 Swings” or NYC's “Giant See-Saws”, activate public interaction in underused areas of the city through the installation of the purpose-built play structures. Ping Pong tables and interactive splash fountains are other common PLLs that, “blend learning, placemaking, and community cohesion for cities” (12). To this, we add skateparks as spaces of grey play, a kind of PLL.

The intent of our paper, however, is not to promote designing more city-built or “official” installations for play. This is not to say that there are not methods for city-built installations that readily elicit skate play, such as the grassroots methods found in skate friendly cities like Bordeaux, France and Malmö, Sweden (13). Our focus is instead on already existing city structures for their propensity for street play by skateboarders, urban citizens that have developed skills associated with their city craft on a wide variety of concreted surfaces (14). Engaging in a subculture devoted to a wooden, wheel-driven, toy, skateboarders learn to be experts of grey play, producing surplus value for the city and its citizens. To support this claim, we consider the variety of skate play in the city, its relationship with capital, law, and significant risk and reward for social identity and human fulfilment. We turn now to a general discussion of play, its why and what for.

## 2 Methods and materials

This research builds off of ongoing conceptual and ethnographic work of various skateboarding scholars that fuse of their own academic paradigm with their skateboarding praxis. For instance, the skateboarding ethnographer Duncan McDuie Ra (7) draws upon his own positionality in skateboarding to make engaged and reflexive claims that otherwise remain invisible to those outside of the skateboarding world. More specifically, McDuie Ra presents a “rolling ethnography” as a valid part of his autoethnographic experience, just as walking, talking, eating, observing and even listening are, to other ethnographers, their most immediate tools and method (15). This follows Maxwell's (16) interactive model, meshing personal goals of qualitative research with a theoretical framework, guided by its preliminary research questions that emerge from our skateboarding practice. Our research thus speaks to a critical engagement with the skateboarding world that we are each a part in our respective cities. Each of us utilize the city for play in many of the diverse ways discussed in this paper, finding a depth of fulfillment in this process, providing a common ground for our analysis that equalizes our differences. This was facilitated by our face-to-face meeting at the skateboarding conference, Slow Impact, in February 2024, Tempe, Arizona, USA where these ideas were both discursively practiced and discussed.

Furthermore, our interdisciplinarity weaves in epistemological assumptions informed by catalogue of theoretical research and study in our individual specialization: philosophy, economics, and anthropology, respective of author order. Similarly, as active skateboarders for decades we are oriented to and informed by the world through our shared activity, making our work a synthesis of situated autoethnographic forms of knowing. Thus, this research has unfolded as an endeavor to comprehend skateboarding, its people, places, and possibilities, constantly observing, discussing, and debating the contexts we are exposed to and wrestling to fathom. In particular, this paper is the fruit of meeting together for skateboarding, discussing, and theorizing on the meaning of skate play—thinking and doing at once.

Our method involved keeping notes, critically discussing these notes, and making site visits to both skateparks, DIY skate spaces, and skate spots in reflecting on their differences and similarities in provoking different forms of skate play. From these site visits, notes, and reading of philosophical, economic, and anthropological texts, we constructed working drafts of essays and pursued an organic form of thematic hand-coding based on our notes and observations. This coding is freeform, focused on linking ideas, and generating themes of reflection, including critical discussions with peer researchers on play. This coding act is quite similar to our shared skateboard “sessions” where various tricks were performed on obstacles in a “line” that links them together, with fellow skateboarders shouting encouragement and being inspired, skate their own line. Thus, our methods are best framed as ethnographic interpretative and philosophical, mirroring the activity that is the object of our research.

Conceptually, our main analysis of skate play and its pluralities follow O’Connor's (9) argument of reducing skateboarding and its various activities to material and symbolic meanings of urban life, what is termed “grey space”:

[T]o make the complexity of skateboarding accessible. In doing so it opens the opportunity to bond elements of research on skateboarding to other lifestyle and action sports, to offer researchers a means to circumvent the contested sportification frame of skateboarding scholarship, and to connect with a variety of disparate scholarly realms (9).

Grey space helps capture the material necessities and symbolic uses of the environment by skateboarders by reducing its meanings to various forms of material and symbolic pollution that inform the hazardous conditions of skateboarding (4) and provide the means for its dangerous play. To this concept of grey space, we add the term “grey play”,^1^ play that is dependent on polluting materials like concrete as symbolically polluting the city, disrupting its common commercial uses of space in various forms of skate play. The grey play of skateboarding is polluted play.

## 3 The processes of play

Theories of animal play are plentiful, helping to understand not just human play, but skate play in the modern city. It is generally accepted that play serves a creative function. “Play enables individuals, after they have sampled their environments, to generate, in a rather low-cost manner, a repertoire of innovative behaviours that may be adaptive to their specific niche” (17). Classic theories of play often connect play to this creative function. For instance, Schiller's theory of the human “play impulse”, is a drive that integrates reason and the senses. Spencer's theory for both human and animal play suggests that play keeps underused capabilities tuned (18). Contemporary theories of play often orbit necessary and sufficient conditions, often involving “play fighting”: conditions that lend themselves to social development, including communication and predation skills within an animal group (19). Cognitive and neurobiological bases for play, particularly in rats, reveal that opioid neurotransmission is the central modulator for social play (20). A healthy animal, whether in the wild, a pet at home, or a subject in a lab, is a playful animal (21). Humans, both youth and adults, also benefit from play, and in fact would be thought unhealthy if they did not (22).

Human play has been described as a socio-poetic engine often imbued with norms, formalizations, and regulations that constitute a “frame” (23), within which individuals consent to adhere to the artificial rules of formalized games. We can observe this process in human history's increased regulation of leisure time: in the historical transition from shamanism to compartmentalized play within defined spaces and times that oppose times of labour, from informal play to formalized sport and games (24). Play, across cultures, is rarely exempt from regulation: agreement around certain social conventions underpins modern human games and even “pure play” orients itself around learned social structures. Abulhawa (2) recognizes this interchange in skate play in the context of Deleuze and Guattari's (25) “smooth” and “striated” spaces. A city's spaces are allocated specific uses and assigned specific times, a “striated” space that the skateboarder's play “smooths” through its transversing flow of tricks.

This antinomy between “pure play” vs. “serious structure” poses a kind of chicken or egg dilemma. Which comes first is at the centre of a debate up to the 1960's (26). Many contemporary studies on sport have examined the fragile thresholds of game institutionalization, to varying extents reproducing the assumptions of this dichotomy (27). One construal of this debate compartmentalizes serious play as powerful when it follows an imitative, virtual, and ritualized “modality of action” (24), such as shamans play imitating animals. As Caillois reminds us, “the principles ruling various types of games […] are reflected to the same extent outside the closed universe of play” (28). Imitation's structure for play is not about its content but its approach—its mode. Imitating animal fighting is not done on all fours like animals, but on two legs like humans—imitative play fighting is about the activity of fighting.

A resolution to this chicken or egg debate favoured here is that play is not bound by either/or categories of informal vs. formal rule-bound game play, but rather that creative play undergoes phases in its activity, from chaotic freedom to institutionalization (29). A plurality of processes of institutionalization operates in principle as play across time (28). By virtue of this origin, play retains within it an indeterminate space of unpredictability in which play as a culturally creative modality can be used to subvert social norms and mores. It is thus a matter of observing not only the outcomes of institutionalization or archaeologically researching the origins of play, but of analysing the ongoing processes in which creative and playful “modalities of action” (24) are continuously articulated between institutions, grassroots negotiations, and cultural creativity. While ethology, the science of animal behavior, can reconstruct the aetiology of human play and its impact on the human body as distinct from animal adaptation (30, 31), there also exists a continuity between animal and human in play, one often attributed to developmental stages, like childhood, even though it may exist throughout a life course.

### 3.1 Neoteny and surplus value

In the context of human neoteny, the retention of juvenile features into adulthood, play retains two forms of value to the individual, society, and environment: (1) Play has a direct capacity for transforming maladaptive behavior to adaptive, (2) Play can optimize resource utilization of the environment through exploration (32). In strictly cultural terms, the transition that marked the difference between adaptive and exploratory play in animals to social play in humans is found in a specific singularity of the human species, whose, “sensorimotor behaviors, [ … ] techniques of the body, are all essentially supported by a material culture without which societies cannot exist” (33). Human play is also distinct from animal play in how humans produce, adopt, consume, and manipulate material objects, creating a material culture that feature learned techniques of the body (34). Human play, thus, derives from the combination of material culture and techniques of the body, also giving rise to risky behaviours central to cultural development.

Contemporary anthropology of material culture and mass consumption reminds us that even seemingly trivial objects, mass-produced and purchasable, when placed in a certain cultural or relational framework, and when observed through the micro-practices they evoke, have a role in the development of subjectivity, in understanding society and its environments. Instead of considering interaction with artifacts produced by capitalism as superficial, frivolous, or ideological, Miller (35) reminds us that there are good reasons to seek, in the manipulation and play with its products, the basis for the social creation of a “self”. Artifacts are thus central in the “process of social self-creation [ … ] directly constitutive of our understanding of ourselves and the others” (35). In this sense, consumer societies are not so different from primitive ones; both develop cultural patterns through interaction-play with a different material culture.

These theories of play are of fundamental importance for the study of skate play: which invokes culturally specific conceptions of the environment, motor skills, and conceptions of surrounding society, such as masculinity (36). Through play we uncover our habits, pleasures, personal abilities, and values, satisfying creative urges embedded into the survival of our species. As much as any other creature, we have been conditioned to be playful to live. Through play, we uncover and establish valuable relations with processes of nature and with the others (37), inside and outside our own cultures and sub-cultures.

The results of this recognition of human play as continuous with animal play, but also distinct in that humans have a material culture, suggests multiple forms of play processes. We argue, for instance, that skateboarding play results in multi-domain effects amongst both individuals and social groups due to its diverse forms of value creation with nuanced multi-use urban resources. With sufficient opportunity, skaters can generate activity in spaces which are not designed for, and may in fact be hostile to, play. From this perspective, skateboarders are icons of crafted play, making spaces and times for their manifest creative tendencies. On even the most banal urban architecture such as stairs, curbs, ledges, etc., skateboarders create value across material, social, political, economic, and cultural domains. Even equipment designed for work, like a backhoe, becomes an object of play (1).

## 4 A plurality of skate play

Tyshawn's manoeuvres discussed in the introduction are comparable to similar feats by other street skateboarders in other cities which elicit all kinds of responses. Some of these responses are well-discussed in skate studies, what we call “common” play and some “uncommon”, to which this section provides a review. Briefly, it is common to discuss how skating in the car-filled streets is extremely risky, placing the skater as well as other pedestrians in significant danger. Thus, some theorize this street play as a kind of “deviant leisure” (38), a “skate crime” (39), and a transgression of social norms (40), one that can sometimes contribute real “social harm” to society (41). Also, it is common to note that Tyshawn is also a professional skateboarder; his play is labour, a “serious leisure” (42). He is like so many other professional skaters whose media output profits corporations, resulting in exploitation and alienation from their labour (43). Thus, we find skate play centred on a conflicting dynamic of “counter cultural” identity for *mainstream* consumption (44).

### 4.1 Common categories of skate play

**Figure 2:**
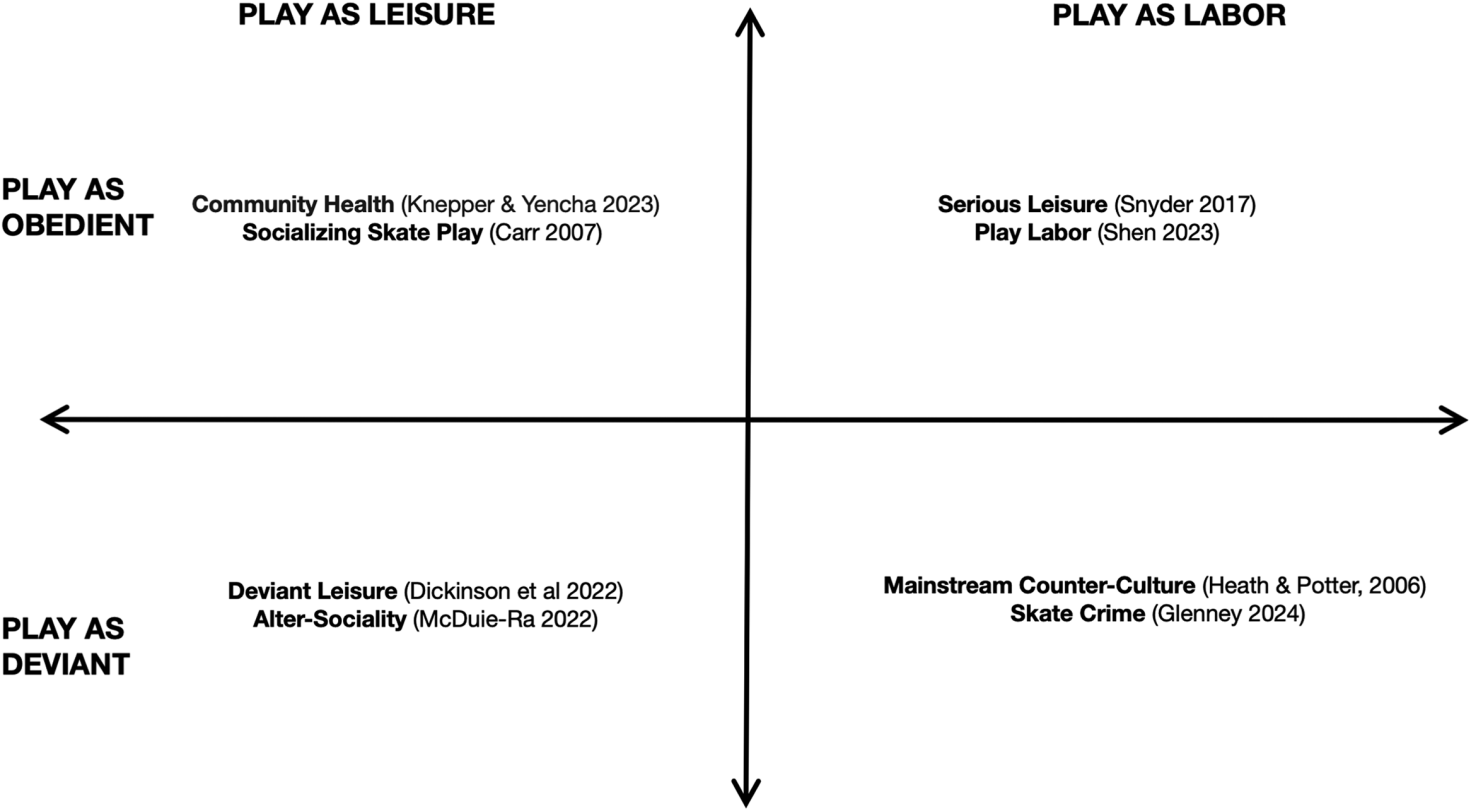
This table charts four accounts of skate play commonly discussed by skate scholars referenced in a Cartesian map. These familiar accounts are distinguished by opposing categories of labor vs. leisure and obedience vs. deviance. These categories are often structured by their association to capitalist forms of production, or lack thereof.

There are many ways that skateboarders play in the city. A closer look at the various views on skate play discussed by skate scholars can be distinguished by familiar categories (see Figure 2). These categories of skate play contrast leisure with labour and deviance with obedience, categories informed by capitalist structures and fitting within common socio-political meanings, norms, and mores.

#### 4.1.1 Deviant leisure

Dickinson et al. (38) and McDuie-Ra (45), refer to skate play as “deviant leisure”, play that deviates from lawful regulations. These deviations subvert social norms using the property of others or public space in a way that is out of compliance with various municipal codes that control the spatiotemporal routines of normal private and public property use, creating a new “aesthetic order” of the city. Similarly, McDuie-Ra (45), argues that skateboarding's deviant play constructs an “alter-sociality” of urban infrastructure (45). Skateboarders’ deviant uses of city architecture are not for purposes of social or political value, or even efficient transportation (46), provoking instead an alternative aesthetic. For Dickinson et al. and McDuie-Ra, skate play produces a kind of apolitical agitation, their deviance is *about* their leisure, viewing the city as a “delinquent playground” (45) [see also (47, 48)]. In sum, the city provides disgruntled skaters play space for a deviance that is for the sake of being deviant.

#### 4.1.2 Serious leisure

Skate play is also sometimes “serious leisure”, not only for play but for financial reward, “from which a large number of people profit” (42). We take Snyder's notion of serious leisure to be akin to “play labor” (49) found in the exploited play of many video gamers. The serious leisure of skateboarding exists within a capitalist framework associated with employment and is thus grounded on exploitation as well as alienation from one's product, such as the film medias that are a central product of skate labour and the “business” of skateboarding (50). Additionally, some play labour includes “sport” contest formats and corporate-sponsored projects which ledger strict bureaucratic limitations, managerial pressures, and cultural expectations on serious forms of skate play (51). Serious leisure is contradictory to the category of deviant leisure skate play, but both play significant roles in skate culture and the lives of individual skaters.

We also distinguish these critical aspects of serious leisure from its more developed form by Stebbins (52) for whom serious leisure has properties that remain joyful in its “cultural richness, notably its shared goals, problems, values, experiences, and costs and rewards” (52). Part of this joy emerges also from participant individualism, agency, and self-determination. “It is evident that the serious leisure participant is for the most part his or her own boss” (52). Clearly, though serious leisure and its play are embedded in a capitalist framework, there's a depth of both social impact as well as its individual benefits that we hope future work in skate studies will expand on following Stebbins (53).

#### 4.1.3 Mainstreamed counterculture

Skateboarders’ play as labour includes Tyshawn's videorecorded manoeuvres, which earn financial benefits from the use of the property of others, selling media through skate and street wear companies as a “counter cultural” act, utilizing the same capitalist logic that makes such acts illegal (44). In fact, much of professional skateboarding involves a variety of “skate crime” (39) that enriches a skater's estate though the appropriation of the property of another. Both forms of play as labour capture aspects of Tyshawn's street play, revealing some of its social density and confounding nature.

#### 4.1.4 Community building

A final form of skate play includes its co-optation by municipalities in reserved city-built sites called skateparks (54). These reserved spaces are a part of a city's urban planning, helping to deal with the nuisance of skateboarders on city streets and sidewalks, attracting them to these recreational areas of supervision and “safety”. Skateparks are zones for learning mainstream social and political values, which according to Carr (36), includes dominant gender roles as well as strategies to subvert them. In addition, skateparks provide space for community and have significant individual social and mental health benefits (55, 56). As Clark and Sayers (57) note, some skateparks in the UK saw a “gender reordering” during Covid and subsequently skateparks were reimagined and spaces of “creative possibilities for recovery” (57). New communities were formed and continued activism in these communities has resulted in more significant recognition for women and transgender skaters of various ages and vocations (58, 59).

This collectivism of skate play at skateparks provides a relief valve for cities with a high density of street skateboarders while also contributing to the health of the skateboarding community. In fact, these reserved spaces of play may mirror the “hydrarchy” of other small-space collectives: ships in sailing seas, small island populations, and communes (60). These spaces offer a socio-political density that provides a context for novel forms of “uncommon” skate play.

### 4.2 Uncommon categories of skate play

Some forms of play by skateboarders are untidy, discomfiting to the common distinctions of leisure/labour and deviance/obedience that help group prior research on play in skate studies. Hence, these kinds of play are less discussed, though Abulhawa (61) has begun to develop an account of one form of these uncommon kinds of skate play: “play for play's sake”. To this we add other forms of play mentioned in other studies on play, such as the gambler's “deep play”, the mountain climber's “edge work”, and a kind of Marxist form of play that produces freedom through labour that we call “Promethean play”. Each form is discussed and grouped by their relationship to risk vs. creativity or exploration and unskilled or freeform play vs. skilled play (see Figure 2).

#### 4.2.1 Causa ludendi

Tyshawn's manoeuvres discussed in the introduction also exemplify a kind of play difficult to define: *ludere causa ludendi,* play for the sake of play (61), an instinctual activity seemingly shared by many non-human animals who have surplus resources (62, 63). While *causa ludendi* tends to resist popular analysis due to fundamental ambiguities—we do not know what *causa ludendi* is for, or why animals, including humans, do it, remaining a mystery in play studies (64)—ethnographers and ethologists continue to view it as a causal force for play, fundamental to understanding our species (24, 62).

The mysteriousness of *causa ludendi* may help account for a similar description of skateboarding as a mystery (65, 66). Skateboarding resists categorization and shows resilience against sportification, capitalist forms of labour, and other forms of mainstream control (51), while also influencing mainstream culture with a counter-culture mystique (44). This *causa ludendi* form of play may be the source of its “sui generis” status that resists analysis, maintaining both its mysteriousness and power.

This *causa ludendi* play appears instinctual and is thought to be common across many species of animal. Bees voluntarily roll wooden bee-size balls backwards presumably because it feels good to do so. What's more, bees who played decreased their amount of foraging time, seemingly trading play for labour (67). Young ravens also played with experimental moveable objects, apparently for no reason other than emotional and social benefit of being with others in ease (68). Beef cattle commonly play with one another, though ceases after being handled (69). Anecdotal stories of animal play is common, including home videos, such as a crow surfing down a snowy roof on a plastic lid (70). These examples suggest various animals in all kinds of spaces have natural tendencies toward play for the sake of play.

#### 4.2.2 Deep play

Yet skateboarders’ street play, exemplified by Tyshawn's cross-traffic tricks, appears unlike the fun play of *causa ludendi*. Less exploratory and curious and more dangerous. Skate play is also a “transgressive” play (71). This dangerous play is like what utilitarian philosopher Jeremy Bentham (72) termed “deep play”: the play of a high stakes gambler going all in. Bentham argued that deep play was so risky with such little reward, that it was immoral; deep play is a social bad in its lack of utility for all. And yet, deep play is prevalent and pernicious in societies throughout the world despite its related unscrupulous manifestations. For instance, gambling on cock fighting manifests deep play (73), giving a propensity to produce irrational bets on bad odds for all who play, “In genuine deep play … they are both in over their heads” (73). By contrast, shallow play is elicited by not just smaller bets, but less risky bets, bets that largely produce thin benefits of utility and happiness. By contrast, for all its non-utilitarian consequences, deep play elicits thick concepts, “much more than material gain: namely, esteem, honour, dignity, respected—in a word … status” (73).

#### 4.2.3 Edgework

While skateboarding manoeuvres are a gamble, like Tyshawn's risky skate play, they are also embodied. These voluntary risk-taking acts have a significant sensory valence and appear to be a structural characteristic of a modern society with greater resources for leisure that allow individual skill development—termed “edgework” (74). Edgework involves sensory, spatial, and skilled mastery of body techniques for navigating complex terrain, like climbers’ vertical movement up rock faces (75). In addition, climbers are known to engage their complex terrain without safety equipment, a parallel with the irrational gambling in cockfighting described by Geertz (73) that generates thicker benefits of both individual and social worth and value. Edgework is a gamble, a deep play that takes on a skilled body-centric focus, a means for spatial appropriation of otherwise hostile terrain for purposes of self-mastery and mastery of an environment, a “sensation-centric locomotor play” (71).

This combination of edgework and deep play seems manifested in skateboarder's city play, as exemplified by Tyshawn's unique and dangerous uses of urban architecture. Skateboarders treat the city—its spaces and socialities—as its sandbox. What's more, this street play has been situated in the context of prefigurative and socio-political imaginations that shape new moral norms and socio-economic structures (76), breaking bifurcations (8), offering novel ethical, economic, and political ways of being, adding value to the city and its citizens. So too, more curious and exploratory kinds of skate play, *Causa ludendi*, invite original uses of the body, city spaces, and social interactions, generating a skater's sense of self and space that orbit new modes of self-expression and self-discovery, a common theme in studies of skateboarders around the world (77). This invites another potential form of play: “Promethean play” (see Figure 2).

#### 4.2.4 Promethean play

Some play fosters self-discovery—a form of exploratory play like *causa ludendi*, but one that is rooted in the exploration of the self and attempting to transcend its assumed limits, often through laborious application of technological achievement (78). Promethean exploratory play often doubles as a domination of one's environment. Such skate play “takes over the city”, and makes it its own. Promethean skate play may thus be exemplified by DIY skateparks, that exert a kind of squatter's rights—a “built to own” mentality that subjugates spaces for its own purposes of creative exploration and freedom of expression, as well as other Promethean virtues: “authenticity, individuality, self-sufficiency, strong commitment” (79). These values cultivate the flourishing of humanity as a perpetual creative force. As Marx writes, “a continuous self-transcending act of coming-to-be” (78). Street skating appropriates city spaces for its own purposes, albeit temporarily, another space ripe for Promethean play. For instance, a sign pole bent over by a hit vehicle is repurposed as a “pole jam” for skateboarders, who use it like a ramp to grind up and over (80) Skateboarding and its Promethean forms of play are a productive force, adding surplus value to the city, creating new technologies out of old ones.

**Figure 3:**
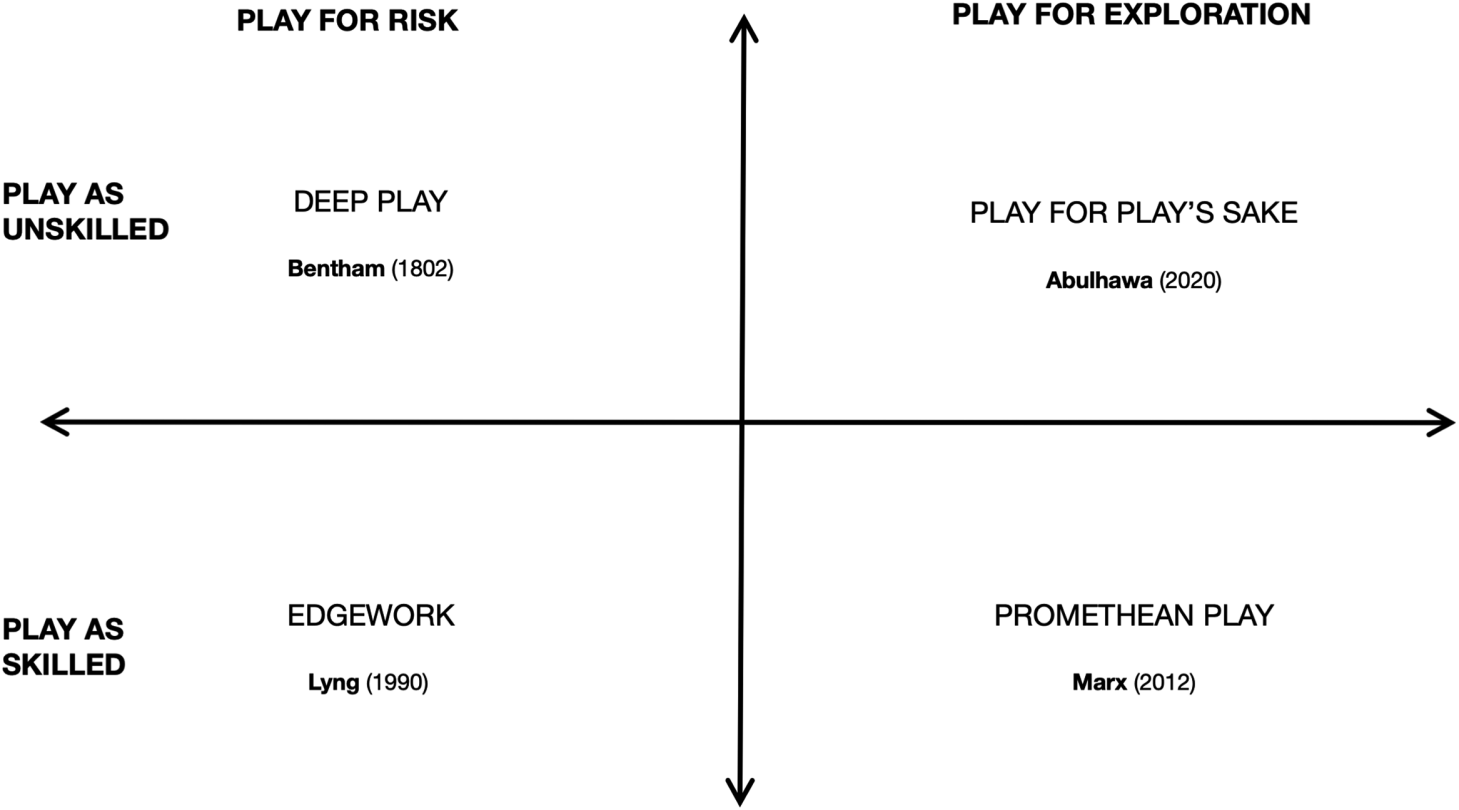
This table charts four accounts of skate play that are not commonly discussed by skate scholars but are associated with the theoreticians referenced in a Cartesian map. These uncommon accounts of skate play are not structured easily due to their irregular socialities but can loosely be distinguished by opposing categories of risk vs. exploration and unskilled vs. skilled forms of play.

These uncommon views on skate play, summarized in Figure 3, are comparable to play in the city without a skateboard. For instance, Grace (81) compares skateboarding, parkour, and skywalking in terms of their risky play and socially beneficial outcome of autonomy. “Risking your life for something you desire allows you to assert your control” (81). These risky acts in the city help participants find a kind of individual agency within a social context of play (82). So too, skateboarding's subversive play and co-optation of space bears a likeness to other urban activities, from roller skates (83) to parkour (84) to graffiti vandalism (85).

In all these forms of play, both common and uncommon, the skater makes use of the surplus resources of urban society (non-policed concrete pavements and factory-manufactured toys) to engage in creative ideas and generate new values—a surplus value. In sum, whether dangerous and transgressive or curious and exploratory, skate play elicits a potential surplus value to the city, creating potential salubrious spaces from dysbiotic concrete surfaces.

## 5 Spaces of skate play

There are many ways cities plan for play. Purpose-built Playful Learning Landscapes (PLLs) like seesaws and swings such as Montreal's “21 Swings” or NYC's “Giant See-Saws”, activate public interaction in underused areas of the city through the installation of the purpose-built play structures. Ping Pong tables and interactive splash fountains are other common PLLs that, “blend learning, placemaking, and community cohesion for cities” (12). Skateparks are another form of PPLs designated for play on wheeled vehicles (80).

We focus on three general types of skateboarding spaces for play as case studies and the diverse kinds of play they encourage:

1. Skateparks: city-built architecture for the purpose of skateboarding encouraging obedient leisure play and play labour.
2. DIY Skateparks: city-built spaces that are manipulated by industrious skateboarders by adding material structures that encourage deviant leisure and uncommon forms of play.
3. Skate Spots: pre-existing material architecture like stairs, handrails, and ledges that are co-opted for deviant leisure, play labour, and uncommon forms of play.

**Figure 4:**
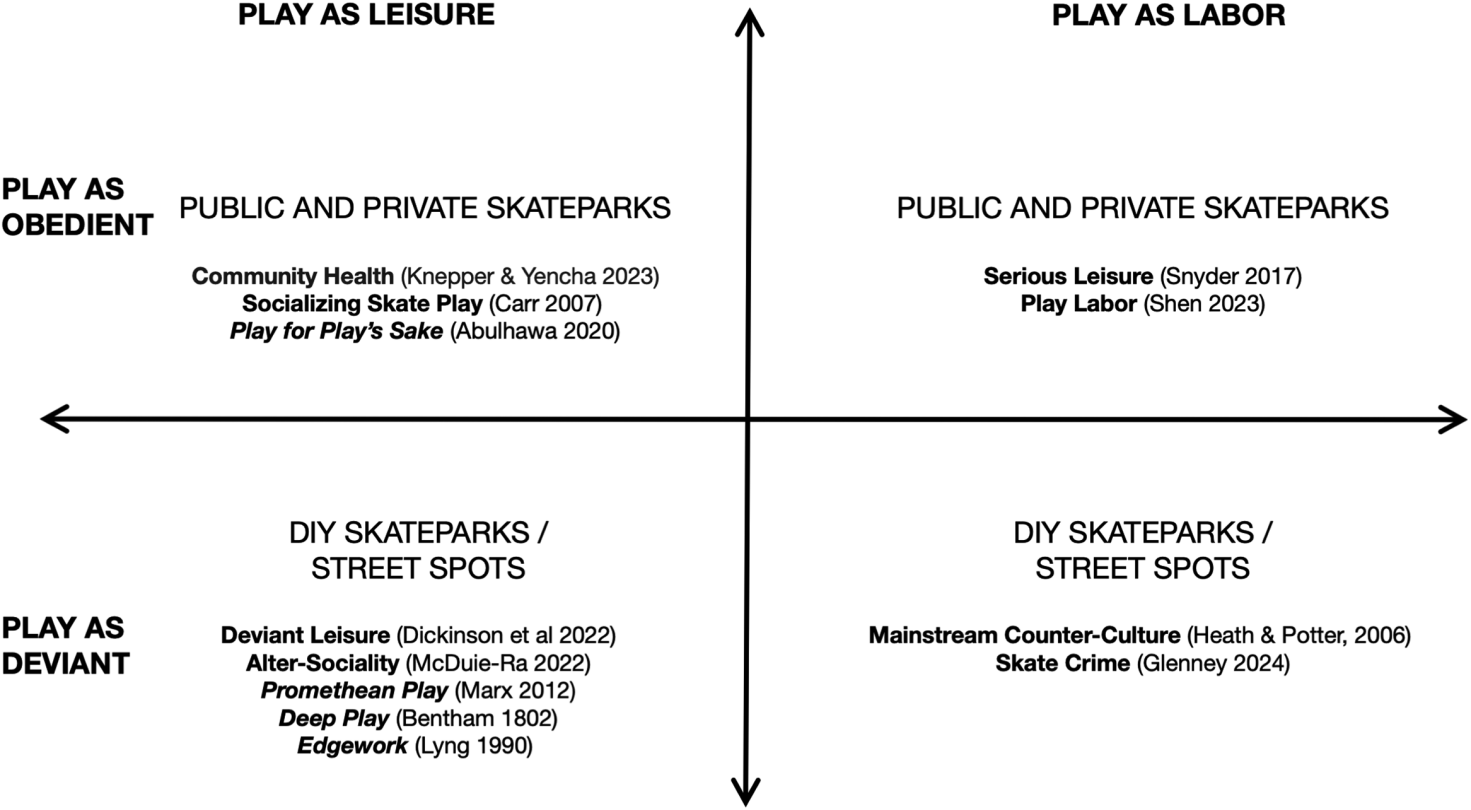
This table charts four common spaces where skate play can be found, as well as both common and uncommon categories of skate play referenced in Tables 1 and 2, in a Cartesian map. As these accounts of skate play spaces use commonly discussed forms of skate play, but includes uncommon forms of skate play as well, an attempt is made to fit the uncommon kinds of skate play with their associated spaces. This suggests that we should expect to find both common and uncommon forms of skate play in the same spaces, particularly in DIY Skateparks and Street Spots.

The city is a dense multi-use space for not just the capitalist regimes for which they were built, but for the production of spaces for all kinds of leisure acts, from recreation, to leisure, to play (see Figure 4).

This focus on skate play in its grey spaces involves three kinds of skate spaces and their diverse play. In the first case, we consider “skateparks”: urban architecture that is designed and built by the city *for* domestic play, comparable to city-built playgrounds, sporting arenas, and “Playful Learning Landscapes” (PLL's). Our second case is “DIY” skateparks: spaces in which skateboarders themselves take direct action in creating their own skate obstacles and illegally occupying spaces. Finally, we discuss coopting already emplaced urban architecture for skate play known as “street skateboarding”. These coopted spaces, known as “skate spots”, are designed and built for commerce and wayfinding by the city, and thus protected for these uses by laws and regulations which are sometimes enforced by citizens, private security, and police (86).

### 5.1 Skateparks and obedient leisure

Most of skateboarding today is conducted in facilities explicitly designed for skateboarding: both public and private skateparks (80). Many skateboarders skate routinely at their local skatepark, an inviting and accessible space, where community and learning are part of a skater's day to day experience (87), particularly for women and minority skateboarders (57). These spaces are known to produce positive physical and mental health benefits (56). Skateparks provide a space that fulfils the recreational intentions of architectural designers and urban planners rather than contradicting them in the streets (88).

Skateparks are typically built in the street's image, with more modern skateparks explicitly labelled as “plazas” replicating real street spots (80). Common skatepark obstacles include replica stairs and handrails, curbs and planter ledges, banks, and empty pools. As skateboarding progresses, the regular obstacles of street skateboarding have shifted, and skateparks have followed suit including “pole jams”, ride-on ledges, and steep brick banks.

The kind of play most often associated with skateparks is like other city-designed/built recreational spaces—for leisure. Skatepark play is obedient leisure as opposed to the deviant leisure associated with skateboarding's co-optation of city architecture (38) or the alter-sociality it invokes (45). Skateparks, like other outdoor public recreation spaces (89), also establish urban oases for skateboarders to play together safely, building community with each other without the challenges offered by the street, though challenges remain (90). Obedient leisure fits within capitalist structures, as it abides by social norms while also being defined in terms of labour and employment—leisure is what one does after work. Hence, skateparks are organized by a municipality's parks and recreation departments, and sometimes considered a “sports facility”.

Another basis for claiming that obedient leisure play is frequently found at skateparks is that their intention and design is to attract street skateboarders from city spaces into their contained reserved facility (54). Local municipalities tend to be in favour of skateparks either as a method to keep skaters out of private property conflicts, or as a component of renovating polluted city spaces like brownfields and Superfund for recreational facilities. Glenney (8), often with the help of funding and branding from major corporations.

Skatepark play is also associated with play labour. Though skateboarding media frequently eschews skateparks for its various medias (50), skateboard contests are often held at skateparks for financial reward, giving skateparks their additional “sport” meaning. Skate play is often viewed as a “sport”, formalized through various rules for contests to rank “winners”. Inclusion in the Olympic Games has also increased the sportification of skateboarding (51).

Lastly, while not directly encouraging some kinds of uncommon skate play, skateparks function as learning spaces for development of bodily skills into the kind of mastery needed for the more dangerous “edgework” (75) of urban spatial reappropriation. “Edgework requires vast amounts of time, money and dedication in *safe spaces* where skills and knowledge can be acquired” (75). Skateparks largely serve this purpose to skateboarders—a safe space where trick skills can be obtained to press into new terrains in diverse ways. It is thus likely that the more creative and exploratory form of skate play, play for play's sake or *causa ludendi*, is elicited using skatepark spaces.

### 5.2 DIY skateparks and deviant leisure

Despite numerous city-built skatepark developments, skateboarders continue to design, build, and occupy DIY skatepark spaces (91). Within these developments, a variety of spaces have come to be publicly acknowledged as space for skateboarders without becoming an official city-supported skatepark. Some of these spaces are built illegally, hidden under bridges and other spaces of urban infrastructure. Some of them are even built under “occupation” and as a challenge to various forms of tyranny and violence. “Skateboarding is considered a practice that embraces a transnational urban playful culture that achieves a subtle undermining of social and political authority wherever it operates” (2). These DIY spaces directly embrace skateboarders’ own choice of design, resulting in more creative skate play in their *own* space.

D.I.Y. skate parks provide skaters with a space where they are encouraged to move architecture around, furthering the reimagination of concrete obstacles and play possibilities (92, 93). Obstacles like parking blocks, portable ramps, and handmade boxes are often charity constructions built by local skaters resulting in creative play. Unlike skateparks and even street spots, as we discuss below, these portable objects generate mobile creativity for skate play, establishing an environment where the possibility of playing with pavement is maximized. The freedom of arbitrary object play is commonly noted to increase playfulness in general playground environments from bees (67) to babies (94). Thus, it is no surprise that a subculture centred around playing with concrete would embrace the opportunity to physically reorganize objects that are typically unmovable, fostering a kind of transgressive recreational behavior (40).

DIY skateboarders are experienced practitioners of playful direct action, a form of pre-figurative politics that aligns with uncommon forms of skate play. Here, individuality is not only cultivated through skate play, but the creation of the obstacles for skate play to “disrupt the fixity of neoliberalism in urban spaces” (91). DIY skateparks offer a design-build ethic of spatial manipulation that promotes new forms of cooperative efforts and turns skateboarding into a maker-culture with a micropolitics. As Manning (95) argues, DIY skateparks “invent new forms of existence” while also “making untimely existing political structures, activating new modes of perception, and inventing new languages that speak in the interstices of major tongues” (95).

### 5.3 Skate spots: deviant serious leisure

Urban design is dictated by the needs of capital and its logic, with architecture and regulations that restrict the pursuit of leisure. Yet, skateboarding manifests imaginative uses of material and social urban spaces in the activity of reappropriating spaces for their own play. Even though city-built and DIY skateparks are a vital component of skate culture, they are ultimately built in the street's image, a kind of hybrid of urban architecture and recreational space (80).

Streets are an industrial infrastructure primarily providing support for automotive vehicles to carry and move commodities and safe wayfinding for pedestrians on sidewalks. Street skateboarding plays these surfaces, giving this infrastructure new meaning. Red curbs that indicate a reserved lane double as indicating a smooth sounding grindable sidewalk edge. Broken bicycle racks become pole jams. Planters become ledges and cellar doors become banked ramps, etc. These new meanings that skateboarders produce out of mundane urban architecture creates surplus value from a wide variety of local urban resources, and thus encourages all sorts of uncommon forms of skate play bent on conquering self and city space.

McDuie-Ra (96) defines a “from below” gaze through which skaters, using the media they produce, observe the city and archive its often-unnoticed spaces. These perspectives offer an alternative to the Western ontology of concrete, where buildings are conceived as abstract superstructures to be erected on the infrastructure that is the road (97). In this sense, skaters in the streets are like pirates in the seas (98), who contradict the vision of commissioning empires existing as a formless space of simple connection and the ship nothing more than a container, a floating fragment of “terrestrial order” in transit between land outposts (99). Skaters, immersed in specific contemporary infrastructures such as concrete and roads, expose the arbitrariness of spatial order and subvert it through their practice, whose use of the street is comparable to the use of the sea by the pirates of yore (39).

The deep play, edgework, and Promethean play of skateboarders, one that can seem to exist outside conceptual norms of law and morality, leads us to a broader discussion about top-down infrastructure and the often opaque or seemingly autonomous levels on which they operate. Urban infrastructure is a conglomerate of techniques, materializing a top-down design from city planning, corporate ideation, or industrial use, etc.: a “striated” space. It is a space of technopolitical exercise (100) but also a catalyst for articulated poetics. Looking at the poetics of infrastructures, and not only at their politics, one can speak of the “ontologies” (plural) of infrastructures (101). The entities in question cannot be regarded as independent objects, but rather as networks—material and symbolic ones—that are intrinsically related to specific subjects, knowledge, and imaginaries. These planned networks, perturbed by the raucous play of street skateboarders, exist as interventions to capitalist structures.

Skate play in the street can disrupt the logic of the market, where roads signify safe and solid surfaces to cross or connect capitalist infrastructures. However, the poetics of skate play, particularly its uncommon kinds, remind us that there exists another dimension from below. Additionally, it emphasizes the importance of analysing infrastructure from a cultural perspective, focusing on the everyday micro-practices and discourses that exist both at the core and the margins of these networks (95). Deeply analysed, modern poetics often exhibit a disconnect between global encoding and local decoding mirroring stratified and smooth surfaces respectively (25). This gap gives rise to heterodox interactions and practices around infrastructures (catalysed by poetics from below) which are often labelled as “piracy”, especially in the global South (102), a street piracy of the city (39).

## 6 The surplus value of uncommon skate play

We find uncommon skate play in all the spaces of more common forms that fit on a labour/leisure and deviant/obedient matrix. Uncommon forms of skate play, and their urban value go beyond a distinctive set of categories. They suggest that, following Marx (78), there are many other ways to value labour beyond exchange value. For skateboarders engaged in uncommon forms of play are acutely aware of the potential of their play to become “play labour”, particularly as their playfulness develops into an exploitable “sport” where their value of labour is largely economic, exploiting how this challenging form of play is almost always somewhat aggravating, dangerous, and damaging to the body.

While economic discourse often interprets Marx's theory of labour value as fixated on a theory of price formation, i.e., exchange values (103), the profundity of Marx's (78) theory of labour value can be found in his younger discussions of unalienated labour and production. There, we find Marx arguing for an uncommon view of human nature that, like our uncommon category of skate play, is not captured by the common categories of labour, play, or human welfare. This form of human nature involves transforming nature, envisioning and executing such transformations, “producing”, seeing themselves as co-creators of their world. This creative relationship is a nutrient of human life and creative power. As Marx writes:

It is, therefore, in his fashioning of the objective that man really proves himself to be a species-being. Such production is his active species-life. Through it, nature appears as his work and his reality. The object of labour is, therefore, the objectification of the species-life of man: for man produces himself not only intellectually, in his consciousness, but actively and actually, and he can therefore contemplate himself in a world he himself has created (78).

Skate play is productive, with kinds of play that lead to self-fulfilment in addition to more common forms of skate play: there are all kinds of “grey play”. The uncommon forms of play of skateboarding involve a spectrum from Promethean, edgework and deep play's seeming necessary compulsion to more exploratory forms like *causa ludendi*. Promethean, edgework, and deep play include a drive for survival and meaning in life without impending alienation and felt exploitation.^2^ “The starting point for all of the sensations, perceptions and skill requirements involved in edgework is the necessity for immediate action to save oneself from death or serious injury” (104). Edgework and deep play are a kind of fulfilling play insofar as it includes danger and risk of injury and death and its unalienated labour: these forms of uncommon play are hard work.

To Marx, unalienated labour, exemplified by all kinds of uncommon play, particularly the laborious deep play and edgework play—autonomously conducted on behalf of one's own creative urges, is a key to human fulfilment. Through unalienated labour, individuals create their own values, transforming themselves. The self is a specific, most intimate, piece of every individual's relationship with nature, materials and his cultural environment. Play motivates humanity to enjoy learning how to manipulate, and even be manipulated, by our surroundings, ourselves, and our peers. Through play we develop and refine the various uses of our body and technologies into craft. We engage in an interaction with our environment, build imaginative relationships with reality, finding fulfilment as we create our identity.

By contrast, alienated labour, such as play labour and serious leisure of common skate play, places agents in exploitable social relations in which the full potential for the fulfilment and creativity in their labour is less easy to manifest: their play is also a kind of employment. Waged labour is exploitative and alienating in that the product of the employee's labour, their valuable transformation of nature, is extracted from them and sent to the market. The playful fulfilment of their labour is not only marginally exploited through wage negotiations, but the valuable product is entirely redistributed without control by the labourer.

Skateboarding does not significantly interfere with corporate overproduction or colonization. Yet it shines a light on the privatization and concretization of our everyday living space and provides a field guide to regain sandbox opportunities in urban grey space (54). Skateboarding is not an escape from capitalism: it relies on concrete and toy factories. Rather, it reveals inconsistencies in a labour-leisure dichotomy of human lifetime. The labour-leisure paradigm of capitalism that accounts for consumer theory as it exists today in mainstream economic theory discusses the exchange value of labour hours and the consumption value of leisure. The values of a human life course are far more plural and intimate.

Skateboarding is laborious, yet it clearly makes fun out of corporate monoliths in its free labour. Yet, capitalism is the grounds for skateboarding: it needs concrete, wood, metal, and its toy mass produced, but skateboarding also reveals the inconsistencies in the logic of capitol, such as the labour/leisure dichotomy, disrupting the centricity of exchange value. Skaters destroy their bodies for free: they labour for typically no exchange at all. They do this in their free time. Skaters are not merely consuming snacks and seeking passive entertainment but are adventuring around real proximate environments of corporatized space. In doing so, the skater sees a difference between their own spatial awareness and that of the public. Public citizens often fail to see active fun in corporate space. They could, if only they had a skater's “sea legs” (39).

Uncommon forms of skate play, difficult to categorize in a labour/leisure paradigm, are perhaps more relatable to instinctual animal forms of play that cannot be so readily subsumed under a capitalist paradigm. These forms of uncommon play are also not clearly within the bounds of deviance, upset the normative codes and social mores. They are seemingly amoral activities of creative impulse—typically more aesthetic than ethic. Uncommon forms of play parallel sandbox play, where imagined possible moral norms and socio-economic structures can be manifest in activities, perhaps offering novel forms of the ethical, economic, and political. Uncommon play is, in this sense, pre-figurative and creative of culture, both features being attributed to skateboarding as a creative outlet for users and a mode of self-expression and self-discovery (76).

## 7 Conclusion

We have argued that the play of skateboarding in the city is diverse but includes a unique form that constructs the self and its environment, difficult to categorize in terms of both economic categories of labour vs. leisure and societal categories of deviance vs. obedience. Thus, uncommon play may be a form of grey play that subvert capitalist forms of alienation and exploitation. Uncommon skate play is a DIY form of identity construction that follows the skateboarder's own craft of co-opting urban architecture for the sake of play.

The transformative capabilities of skateboard play reimagine the resources of urban space from the dysbiotic to the salubrious. Concrete, cement, asphalt, and various hard and flat industrially manufactured surfaces occupy the city, creating barriers that prevent play in green and blue spaces. Few animals have adapted to live in these spaces which have replaced their natural habitat, a habitat wherein humans evolved their forms of play. And yet, as argued here, skateboarding enskins users with the ability to transform these surplus resources of urban architecture and public space into valued landmarks called “skate spots”, and other related spaces that include DIY building practices (8, 105).

Various urban planning strategies for restoring urban health, such as greening cities or designing for biodiversity (106), have come under serious criticism—a green monolith is still a monolith (5). In fact, these greening strategies add stultifying “anti-play” codes and bylaws, hostile architecture, and surveillance and security. In terms of surplus resource theory of play (62), these “greening monoliths” can still limit the opportunity for valuable connections to be made through playful transformations of property relations. By contrast, during the lockdown of the C-19 pandemic, many of these codes and bylaws were not enforced by active security, leading to “hockey-stick” growth in skateboarding and a noted enlivening of urban space (107). Skateboarding is a kind of play that benefits most when the protections of private property are weak, and the urge to actively play is strong.

When city planning tactics control the uses of urban spaces too tightly, preventing play and its adaptive benefits, even with intentions of diversifying its spaces, its urban spaces seize up and stagnate. The way to improve the salubrity of the city, we conclude, is to not just open spaces materially, but opening spaces socially for citizens to comfortably and naturally initiate frames of play (23). At least, this is how skate play creates salubrity from urban space. Thus, skate play in its diverse common and uncommon forms links with Fitzgerald (5) as, “a glitch in these deathly visions [of the city] … something unexpectedly grey and graceful, something clean and sharp and new, [to] cut through the landscape, and a timeline, that we have now been too long given to imagine as unchangeable, eternal, inevitable, natural” (5).

## Data Availability

The original contributions presented in the study are included in the article/Supplementary Material, further inquiries can be directed to the corresponding author.

## Footnotes

^1^The term “grey play” was first coined by Dani Abulhawa and publicly mentioned by collaborator Harry Meadley at “Common Grounds” conference, hosted by Esther Sayers on May 16–17, 2024, London, UK.

^2^In Marx’s abstraction we are imagining labor sources under no coercion, a scenario that is hard to imagine in today’s world where nearly all life is transactional and subject to alienation and exploitation.
